# Identifying and Exploring Sustainability Determinants of Mental Health Recovery-Oriented Interventions: A Mixed Methods Study Protocol

**DOI:** 10.1007/s43477-022-00052-5

**Published:** 2022-08-22

**Authors:** Eleni Sofouli, Shannon Wiltsey-Stirman, Danielle Groleau, Michel Perreault, Myra Piat

**Affiliations:** 1 Department of Psychiatry, McGill University, 1033 Pine Avenue West, Montreal, QC H3A 1A1 Canada; 2 Douglas Mental Health University Institute – Research Centre, 6875 LaSalle Boulevard, Montreal, QC H4H 1R3 Canada; 3 National Center for PTSD, NC-PTSD, 795 Willow Rd, Menlo Park, CA 94025 USA; 4 Department of Psychiatry and Behavioral Sciences, Stanford University School of Medicine, Stanford, CA USA; 5 Lady Davis Medical Institute, Jewish General Hospital (ICFP), 4333 Chemin de la Côte-Ste-Catherine, Montréal, QC H3T 1E4 Canada

**Keywords:** Mental health services, Recovery-oriented interventions, Sustainability, Mixed methods research, Case study

## Abstract

Mental health recovery is the new paradigm in the mental health service delivery system worldwide. Recovery-oriented services go beyond traditional clinical care that is centered on symptom remission, aiming to help people: restore social connections with other individuals and the community; develop hope and optimism for the future; reconstruct an identity beyond that of a “mental patient”; discover meaning in life; and feel empowered to gain control over treatment (CHIME framework). Over the last ten years, several efforts at implementation of recovery-oriented interventions have been documented in the scientific literature. However, little attention has been given to their sustainability, even though it is reported that not all health interventions can fully sustain their activities beyond the initial implementation phase. The aim of this mixed methods case study is to better understand the factors that determine the sustainability of two recovery-oriented interventions (peer support and recovery training) after their roll-out in four organizations in Canada that provide community housing for adults with mental health challenges. Qualitative and quantitative data will be collected from managers, service providers, and implementation team members that oversaw the implementation process along with organizational documents. Data collection and analysis will be guided by the Consolidated Framework for Sustainability Constructs in Healthcare, the Framework for Reporting Adaptations and Modifications, and the Program Sustainability Assessment Tool. Findings will expand our current evidence base on the intersection of sustainability and mental health recovery interventions that remains under-explored.

## Introduction

The transformation of mental health services to recovery-orientation is a policy priority for countries worldwide including Canada (Chang et al., 2021; De Wet & Pretorius, 2021; Piat & Sabetti, 2009; Pincus et al., 2016; Tse et al., 2013). Through the recovery lens, mental illness is seen as only one facet of a person’s life as the person works to recover from the disruptive clinical symptoms of the disorder as well as the wider impact of the illness (e.g., stigma) in their life (Anthony, 1993; Davidson & Roe, 2007). In essence, mental health recovery gives primacy to individual agency, self-determination, and autonomy so that a person can pursue a life according to their values system and aspirations and participate as an equal citizen in all facets of economic and civic life, regardless of the presence of mental illness (Davidson & Roe, 2007).

This conceptualization of recovery is a radical departure from the way mental healthcare was originally conceived, embraced, and operationalized by mental health services for many decades. Traditionally, mental healthcare was centered on treating the symptoms of the illness to prevent further decline of a person’s functional status and/or to minimize the risk to society of their behavior (Lysaker & Lysaker, 2012). In the same vein, engagement with meaningful tasks (e.g., employment) or social roles or relationships that could destabilize the person with serious mental illness was not recommended (Lysaker & Lysaker, 2012). In this context, recovery was understood as the sustained remission of clinical symptoms of mental illness that allowed a person to return to a healthy pre-illness status and be functional in normal day-to-day activities (Davidson & Roe, 2007).

In contrast, recovery-oriented services for people with mental illness are person-centered and aim to promote (a) service users’ involvement in the planning and delivery of mental health services along with self-determination and choice with regard to the type of services and life goals they would like to receive and pursue; and (b) a collaborative relationship between service providers and users of mental health services (Farkas, 2007). The CHIME (Connectedness, Hope and optimism about the future, Identity, Meaning in life, and Empowerment) conceptual framework on recovery provided the backdrop to the key recovery processes that mental health services could work towards restoring social connection with other individuals as well as with the community, fostering hope and optimism for the future, support in reconstructing an identity beyond that of “mental patient,” discovering meaning in life, as well as empowerment to gain control over treatment (Leamy et al., 2011). CHIME was developed through a knowledge synthesis of 97 papers from 13 countries (Leamy et al., 2011). Nowadays, it is used broadly as an exploratory framework for reviews (Jagfeld et al., 2021; van Weeghel et al., 2019; Yung et al., 2021) and individual studies for diverse populations worldwide (Brijnath, 2015; Piat et al., 2017). On the ground, a recent systematic review by Piat et al. (2021) identified seven concrete recovery-oriented interventions: (1) hiring peer support workers (i.e., people with lived experiences of mental illness who have successfully coped with the adversities of the illness and can support other people experiencing similar problems); (2) staff training on recovery; (3) recovery colleges (i.e., educational courses on recovery for the public where courses are co-produced with people who use mental health services); (4) personal recovery planning (i.e., developing treatment plans within service provider-service user collaboration); (5) service navigation and coordination (i.e., supporting users to access services across health and social services); (6) e-recovery (i.e., online interventions on self-help and shared decision making); and (7) family-focused interventions (i.e., supporting families with mental health problems).

Over the past two decades, there has been a growing research literature on the conceptualization, effectiveness, and operationalization of mental health recovery into services (Canacott et al., 2020; Le Boutillier et al., 2011, 2015; Leamy et al., 2011; Piat et al., 2021; Sreeram et al., 2021; van Weeghel et al., 2019). Studies on the different facets of mental health recovery were conducted initially in high-income Western countries and then expanded to other regions (e.g., Asia, Africa, Middle-East) (Sofouli, 2020). However, less attention has been given to the sustainability of recovery-oriented interventions and practices over time. Specifically, this includes the processes that sustain the interventions, factors influencing sustainment, and strategies to facilitate sustainment.

Nugent et al. (2017) in a study of occupational therapists’ perspectives on sustaining their recovery-oriented practices highlighted this knowledge gap. A review by Gee et al. (2017) found only four research projects that examine the sustained effect of recovery training of staff in inpatient settings. While McPherson et al. (2021) in their systematic review on the efficacy of recovery training for mental health staff in supported accommodation services did not find any studies looking at sustained change. Other relevant research has assessed the sustainability of supported housing (Nelson et al., 2017), supported employment (Bergmark et al., 2019), and other evidence-based psychosocial interventions (e.g., assertive community treatment, illness management) (Bond et al., 2012). However, as Oh (2016) and Piat et al. (2021) have pointed out, it is debatable whether some of these psychosocial rehabilitation interventions (e.g., assertive community treatment, illness management) are situated within the recovery paradigm either because these interventions pre-date the recovery paradigm or their impact on personal recovery outcomes (e.g., promoting self-determination, instilling hope) has not been specified.

## The Importance of Sustaining Healthcare Interventions

The integration of a health innovation into routine practice, even if its effectiveness has been proven, generally is a lengthy process that can take up to 20 years (Bauer & Kirchner, 2020). Moreover, research has demonstrated that successful implementation of a healthcare intervention does not guarantee its sustainment over time (Stirman-Wiltsey et al., 2012). In fact, research has shown that a number of evidence-based rehabilitation interventions in mental healthcare (Bond et al., 2012; Stirman-Wiltsey et al., 2012) have failed to be integrated into routine practice of healthcare organizations. Similarly, the positive impact of training clinicians in skills to improve patient engagement in inpatient rehabilitation units diminishes after the completion of their training (Lean et al., 2015).

Unsustainable healthcare interventions have ethical, financial, and political consequences including the lack of long-term effects in population health; expenditure of resources in an era of scarcity and competing priorities; and demoralization of stakeholders (e.g., funders, clinicians) (Lennox, 2020). In addition, unsustainable healthcare interventions create disincentives to participate in future healthcare improvement endeavors, and increased disparity in access to best practices in healthcare (Altman, 1995; Lennox, 2020; Shediac-Rizkallah & Bone, 1998). In mental health, the financial loss due to unsustainability of evidence-based interventions may be enormous considering that in 2011, the global total cost of mental disorders was $2.5 trillion USD, and, with conservative calculations, is expected to rise to $6 trillion USD by 2030 (Bloom et al., 2011).

## Objectives

This proposed research is an initial attempt to address the gap in current knowledge about sustainability of recovery interventions. It aims to better understand not only the “what” but also “why” and “how” of recovery-oriented interventions implemented in community-based organizations for adult mental health can be sustained. For the purpose of this study, we will adopt the definition of sustainability developed by Moore et al. (2017) which includes the following elements: “(1) after a defined period of time, (2) the program, clinical intervention, and/or implementation strategies continue to be delivered and/or (3) individual behavior change (i.e., clinician, patient) is maintained; (4) the program and individual behavior change may evolve or adapt while (5) continuing to produce benefits for individuals/systems” (p. 7).

The study aims to answer the following questions:

1. To what extent have the recovery-oriented interventions been sustained (e.g., sustained, not sustained, partially sustained) after their implementation and once external implementation supports (i.e., research team) were removed?
2. What was the capacity of organizations to sustain recovery-oriented interventions and how did this influence sustainability outcomes?
3. What were the sustainability determinants of recovery-oriented interventions in participating organizations and how did they influence sustainability?
4. How does sustainability capacity and other determinants explain sustainability outcomes?

## Methods

This is a mixed methods case study which combines qualitative and quantitative data to gain an in-depth understanding of a phenomenon in its real context (Creswell & Piano Clark, 2017). Four organizations that implemented recovery-oriented interventions for adults have been selected for study as cases to explore the sustainability of recovery-oriented interventions after external support (the research team) is withdrawn. We will employ a convergent mixed methods design where qualitative and quantitative data will be collected in parallel, analyzed separately, and then merge findings at the stage of interpretation (Creswell & Piano Clark, 2017).

Mixed methods designs are commonly used in implementation and sustainability health research (Palinkas et al., 2015; Proctor et al., 2015; Shelton et al., 2018). Health services are complex systems with continuous dynamic interactions among internal and external actors and other social systems (Greenhalgh & Papoutsi, 2018). Mixed method case study methodology is well suited to such a dynamic, unpredictable, and uncertain environment and can reveal context-specific parameters and their associations (Greenhalgh & Papoutsi, 2018). The implementation and sustainment of evidence-based healthcare interventions is a multifactorial issue that cannot be fully understood by a single methodological approach (Palinkas et al., 2015). By employing multiple research methods, we can offset the limitations of each design (Creswell & Piano Clark, 2017).

Three robust frameworks in implementation science and sustainability research will guide the structure of each research method: 1) Program Sustainability Assessment Tool (PSAT) (Luke et al., 2014); 2) Consolidated Framework for Sustainability Constructs in Healthcare (CFSCH) (Lennox et al., 2018); and 3) Framework for Reporting Adaptations and Modifications-Enhanced (FRAME) (Stirman-Wiltsey et al., 2019). Each framework is briefly described below. Schell et al. (2013) developed a conceptual framework for program sustainability capacity through an extensive literature review, concept mapping, and input from an expert panel. Luke et al. (2014) converted the framework to the Program Sustainability Assessment Tool (PSAT). PSAT assesses the ability of organizations to sustain public health and social care programs against 8 domains that have been found to impact the sustainability of health programs: political support, funding stability, partnerships, organizational capacity, program evaluation, program adaptation, communications, and a strategic plan.

The second framework that will be applied, CFSCH, emerged from a systematic review where the authors synthesized evidence from 62 articles on sustainability frameworks, models, checklists in healthcare, including PSAT, spanning from 1998 to 2017 (Lennox et al., 2018). It comprises 6 domains (the initiative design and delivery, negotiating initiative processes, the people involved, resources, the organizational setting, and external environment) and 40 sub-constructs (Lennox et al., 2018). This framework aims to guide healthcare researchers, practitioners and evaluators to plan for sustainability at multiple levels (system/organization/ program/ intervention) and at multiple points (before or after the completion of the implementation)(Lennox et al., 2018).

Stirman-Wiltsey et al. (2013) developed through a literature review FRAME, the third and last framework that we will use in this study. The original framework aimed at facilitating the classification of adaptations. Authors refined FRAME in 2019 through (a) reviewing the literature; (b) analyzing qualitative data from mental health staff including administrators; and (c) integrating suggestions of stakeholders for modification (Stirman-Wiltsey et al., 2019). The updated FRAME has expanded to allow users to understand the full process of adaptations including eight elements: “(1) when and how in the implementation process the modification was made; (2) whether the modification was planned/proactive or unplanned/reactive; (3) who determined that the modification should be made; (4) what is modified; (5) at what level of delivery the modification is made; (6) type or nature of context or content-level modification; (7) the extent to which the modification is fidelity-consist; and (8) the reasons for the modification, including (a) the intent or goal of the modification (e.g., to reduce costs) and (b) contextual factors that influenced the decision” (Stirman-Wiltsey et al., 2019, pp. 3–4).

Even though the focus of this study is on the sustainability of recovery-oriented interventions, it was deemed important to explore potential adaptations of the interventions for two reasons. Firstly, adaptation is one of the key determinants in sustaining healthcare interventions (Lennox et al., 2018; Luke et al., 2014). Throughout the implementation process, an intervention might be modified, successfully or not, for several reasons. If these modifications are not fully recorded or understood, then this may negatively impact successful implementation and/or evaluation and consequently, the sustainment of the intervention and/or its benefits (Stirman-Wiltsey et al., 2019). Secondly, the type and nature of adaptations will determine the level of sustainment of the recovery-oriented interventions. For example, if the reported adaptations compromise core principles of mental health recovery (e.g., self-determination of user of mental health services), then the intervention would not be considered as sustained since it will have deviated from its initial goal (i.e., implementing recovery).

## Research Setting and Sites

This study builds on from a pan-Canadian project that was conducted between 2017-2021 (Piat et al., 2022) aimed at facilitating and evaluating the implementation of recovery-oriented guidelines developed by the Mental Health Commission of Canada (Mental Health Commission of Canada, 2015). This blueprint for change identifies six dimensions of recovery-oriented practice: (1) Creating a culture and language of hope; (2) Understanding that recovery is personal; (3) Understanding that recovery occurs in the context of one’s life; (4) Responding to the diverse needs of everyone living in Canada; (5) Working with First Nations, Inuit and Métis; and 6) Transforming services and systems to a recovery-oriented approach (Mental Health Commission of Canada, 2015). Each dimension illustrates: (a) key values, knowledge and skills for practitioners to work in a recovery-oriented service; and (b) concrete actions/interventions that organizational leaders could undertake/implement to change the course of their services towards recovery (Mental Health Commission of Canada, 2015).

The initial pan-Canadian project focused on system transformation and the sixth dimension (Piat et al., 2022). The research team recruited seven organizations that provide services to adults with mental health challenges across five Canadian provinces using a rigorous process to ensure cultural, linguistic, and geographic diversity, along with organizational readiness (motivation and capacity) for implementation (Scaccia et al., 2015). Each of the seven organizations selected a service/program where the implementation of the recovery guidelines would take place and formed a multi-stakeholder implementation team (Albers et al., 2020) that went through the same planning process facilitated by the research team. The goal for each implementation team was to select a recovery-oriented intervention based on their local context and needs, and complete an Action Plan that was meant to be an implementation road map for each implementation team (Piat et al., 2022). Each implementation team described in the Action Plan the recovery-oriented intervention to be implemented, specifics (e.g., resources), timelines, deliverables, and implementation strategies to overcome barriers (Piat et al., 2022). The research team continued to support the implementation teams for another 12 months to operationalize their Action Plans and implement their recovery-oriented interventions. In all, 4 recovery-oriented interventions across the seven sites were implemented: 1) recovery training for staff (train-the-trainer model); 2) peer support (Stratford et al., 2019); 3) Wellness Recovery Action Planning (WRAP) (Canacott et al., 2020); and 4) family support groups (Canadian Mental Health Association of New Brunswick, 2021). In December 2020, during post-implementation data collection, two out of the seven organizations reported sustaining their interventions.

Four out of the seven organizations of the initial project were selected as sites for this follow-up study: a publicly funded psychiatric hospital with outreach services and three community-based organizations located in urban centers across three Canadian provinces. These four sites were selected purposefully to attain maximum variation on the implemented intervention and initial sustainment outcome, which was reported in the post-implementation period of the initial project. This sampling strategy will allow us to (a) maximize our learning by including two recovery-oriented interventions and two sustainment outcomes; (b) identify differences in the factors that influence the sustainment or not of the two recovery-oriented interventions; and (c) identify “common patterns that cut across variations” (Palinkas et al., 2015, p. 534). The selection criteria used for the four sites are (1) the same recovery intervention was implemented in more than one site; and (2) the reported sustainment outcome of the intervention varied between sustained and not sustained. The final selected sites include two sites that implemented recovery training for staff of which one reported sustaining the intervention while the other did not, and two sites that implemented peer support with similar results as the former. The two implemented interventions are presented briefly below.

Peer support workers apply their experiential knowledge of mental illness and recovery, “along with relevant training and supervision, to facilitate, guide, and mentor another person’s recovery journey by installing hope, modeling recovery, and supporting people in their own efforts to reclaim meaningful and ratifying lives in the communities of their choice” (Stratford et al., 2019, p. 630). Peer support has been implemented in a wide range of settings (e.g., inpatient units, crisis services, community-based organizations) and peer support workers have taken various tasks including advocacy, practical support, crisis support, etc. (King & Simmons, 2018). In our study, the two organizations that implemented peer support hired three part-time peer support workers to support people with mental health challenges residing in community-based housing. In one site, support took the form of one-on-one sessions with service users, while in the other site, the plan was for the peer support worker to provide more practical support (e.g., accompanying a service user to a doctor’s appointment, etc.).

Staff training on recovery principles is one of many innovations described in the literature that organizations employ to implement recovery into services (Piat et al., 2021). Training/educative initiatives on mental health recovery intend to change staff’s knowledge on recovery, and non-recovery-oriented perceptions and practices (McPherson et al., 2021). The two organizations that implemented recovery training for staff contracted external trainers with an expertise in mental health recovery to deliver the training. In one site, the plan was for staff to attend monthly training sessions over 12 months, while in the other site, staff had planned 13 training sessions over 11 months. The COVID-19 outbreak interrupted the implementation of this innovation, however, both sites resumed training during the pandemic. Both training programs covered a range of topics related to recovery (e.g., differences between personal and clinical recovery, valuing experiencing knowledge of mental illness, etc.), included service users and a train-the-trainer component. Upon completion of the official training, trained staff would take the lead and provide training to other colleagues in their organizations.

## Ethics

The executive directors of three organizations support this research. A final decision from the executive director of the fourth organization is pending. The Research Ethics Board of the Montréal West Island Integrated University Health and Social Services Centre reviewed and approved this research. Study participants will sign a consent form and retain a copy once they agree to participate. Service users participating on the implementation teams will be offered a small monetary compensation to offset their time, if they agree to participate in the study. Staff including managers will participate in data collection activities during their work time, thus no additional compensation will be provided. Anonymity of participants will be respected in knowledge dissemination activities.

## Sample and Recruitment

The sample is designed to explore sustainability through the perspectives of multiple actors (Proctor et al., 2015; Shelton et al., 2018). The goal is to elicit the experiential knowledge from different stakeholder groups and integrate this knowledge in sustainability efforts to increase their success. The sample across sites includes (1) Implementation teams (*n* = 4, maximum six individuals per team); (2) Managers (*n* = 12); (3) Service providers (*n* = 12); (4) Peer support workers and recovery staff trainers (*n* = 4) who were hired to provide the intervention following the implementation of the action plans (Table 1). The estimated sample size is based on the concept of “information of power” in qualitative research; a smaller number of participants is required, if they hold an extensive information power (Malterud et al., 2016).

**Table 1:** Sample sizes and number of interviews across stakeholder groups and study sites

Stakeholder groups	Implementation teams	Managers	Service providers	Peer support workers	Recovery trainers
*Sites*					
Peer support (sustained)	1	3	3	1	0
Peer support (not sustained)	1	3	3	1	0
Recovery training (sustained)	1	3	3	0	1
Recovery training (not sustained)	1	3	3	0	1
Number of interviews per stakeholder group	4	12	12	2	2

Two purposive sampling strategies will be employed: (1) key informants and (2) snowball sampling (Patton, 2015). Key informants or key “knowledgeables” are individuals with a thorough knowledge of a setting who can shed light on the topic of the study (Patton, 2015). In this study, key informants include the four implementation teams formed in the initial study that hold in-depth knowledge on the implementation of the recovery-oriented interventions in their sites. Through their prolonged and active engagement over a 3-year period with the research team, they have developed a shared understanding of the process used to implement their recovery-oriented intervention. They have also learned to identify and tailor strategies to address implementation barriers and “create a sense of mutual accountability for building the infrastructure needed to sustain change” (Metz & Bartley, 2020, p. 199).

Snowball sampling (Patton, 2015) will be used to recruit additional participants for the study. Implementation team study participants will be asked to identify managers and service providers actively involved in the implementation and sustainment of their recovery-oriented interventions. Study participants will be recruited via an email explaining the research project and the related time commitment following an online presentation of the research project in lay language in each of the four sites. The eligibility criteria for study participants are (a) 18 years and older; (b) can understand and write English or French; (c) have experienced the implementation of recovery-oriented interventions at their site or have knowledge about the implementation; and (d) consent to audio recording. Individuals who express an interest in participating in the research will be screened against the above eligibility criteria.

## Data Collection

The first author (ES) will conduct all the data collection activities in the context of her doctoral work supported by her supervisor (MPi) and other co-authors in their capacity as doctoral advisory committee members (SWS, DG, MP). Multiple sources of data will be collected to capture the complexity of the case and provide an in-depth understanding of the phenomenon under investigation (Yin, 2014). This convergent mixed methods study will involve quantitative and qualitative data collection as well as secondary documentation.

### Quantitative Data Collection

Study participants will complete two quantitative questionnaires: (a) a brief socio-demographic questionnaire with questions on age, gender, education, stakeholder group, etc. and (b) the Program Sustainability Assessment Tool (PSAT) (Luke et al., 2014). PSAT has been tested and validated in a variety of health settings and programs. It demonstrates good psychometric properties: (a) subscale reliability ranged from 0.79 to 0.92 with an average of the eight subscales of 0.88; and (b) construct validity analysis—overall score; 0.68 (Luke et al., 2014). Participants rank their programs in relation to the extent to which each sustainability element that was described previously is present using a 7-point Likert scale that runs from “little or no extent” to “a very great extent.” This 40-item questionnaire is user-friendly, available online and can be completed in 15 min. Service providers and managers will complete the questionnaire either in a paper format or online. The toolkit is freely available: https://www.sustaintool.org.

### Qualitative Data Collection

Study participants will participate in focus groups or individual interviews and complete an open-ended questionnaire. Specifically, implementation members will participate in one focus group per site. We will conduct four focus groups in total across sites. A focus group is appropriate since it “capitalizes on the interaction within a group to elicit rich experiential data” that could not be easily obtained through a different method (Asbury, 1995, p. 544). We will conduct individual interviews (*n* = 28) with managers, service providers, recovery trainers, and peer workers who have been involved in sustaining the recovery-oriented interventions. We selected this qualitative data collection method because it will provide rich information from participants that are actively involved in sustaining the recovery-oriented interventions and/or have been impacted by sustainment efforts.

The guides for focus groups and individual interviews will draw upon the Consolidated Framework for Sustainability Constructs in Healthcare (hereinafter CFSCH) (Lennox et al., 2018). The qualitative interview guide will include questions comprising all constructs that were found as influencing the sustainability of interventions in the post-implementation period. These are “available resources, demonstrating effectiveness, monitoring progress over time, integration with existing programs and policies, training and capacity building, stakeholder participation, leadership and champions, organizational values and culture, funding, accountability of roles and responsibilities, belief in the initiative, defining aims and shared vision, intervention adaptation and receptivity” (Lennox et al., 2018, p. 12). A few examples of questions are: (a) Did your organization invest resources in the intervention? If yes, what type of resources? [Resources_General]; (b) To what extent have you assessed or measured the impact of the intervention on mental health service users? [Demonstrating Effectiveness]; (c) Have you taken any appropriate steps to gather and report data for health needs and the intervention’s effectiveness? If yes, could you please explain the steps and the collected data? [Monitoring progress over time].

Interview guide questions will be ordered in a way as to reduce bias and elicit primarily what is salient for the interviewee. Thus, at the beginning of the interview, we will ask the study participants to confirm the present status of the implemented recovery-oriented innovation (i.e., delivered or not) and two open-ended neutral questions to identify and explain factors that determine the level of sustainment of the recovery-oriented interventions. For example: (a) What factors do you think played/play a key role in sustaining/not sustaining peer support/recovery training at (the name of the site)? (b) How do you think that these factors impacted on the sustainability of peer support/recovery training? We will then ask participants the questions on the 10 CFSCH constructs.

We will ask managers, service providers, and peer support workers or recovery trainers from the sites that offered the recovery-oriented intervention after the research team withdrew (December 2020) to answer in writing eight open-ended questions. The goal of the open-ended questions is to capture the adaptations, if any, made to the intervention. The open-ended questions are based on the Framework for Reporting Adaptations and Modifications-Enhanced (hereinafter FRAME) (Stirman-Wiltsey et al., 2019) that was described previously. For example, participants will be asked (a) When did the modification occur? (b) Were adaptations planned or unplanned? (c) Who participated in the decision to modify peer support service/recovery training? etc. Study participants will answer the open-ended questions thinking generally/overall across all modifications made to their innovation as the adaptation of recovery-oriented interventions post-implementation will also be explored in focus groups and individual interviews.

### Organizational Documents

We will collect organizational documents related to sustainability of the recovery-oriented interventions in the post-implementation period. These include progress reports, annual budgets, program events, media releases, and attendance registries of peer support sessions/activities or recovery-oriented training sessions. We will determine the number and type of documents during the research process and assess and select documents based on their authenticity, credibility, representativeness, and meaning (Morgan, 2022). The first author will keep a detailed catalog of the selected documents for each case. Selected documents will be stored electronically. As Yin (2014) mentions, documents “corroborate and augment evidence from other sources” (p. 107), in this case, documents will help authors to triangulate and add context to qualitative and quantitative findings.

### Data Collection Procedures

We will pilot-test all data collection tools in one of the four sites (Creswell, 2013). Any issues identified during pilot testing will be addressed and we will revise the tools accordingly (e.g., questions will be refined to improve clarity). Due to health concerns related to COVID-19, we will conduct all data collection activities remotely.

## Data Analysis

Data analysis will consist of within-case analysis and cross-case synthesis of the findings (Yin, 2014). We will analyze data from each case to provide a comprehensive understanding of the unique issues in each case (Yin, 2014). We will conduct a cross-case synthesis afterward allowing for similarities, dissimilarities, and common patterns to emerge (Yin, 2014). The first author (ES) will take the lead in data analysis supported primarily by her supervisor (MPi), and the other co-authors (SWS, DG, MPe) who have an extensive experience in data analysis. ES has co-authored implementation research articles and has been involved in qualitative and quantitative data analysis.

### Data Preparation

Interviews will be audio recorded digitally, transcribed verbatim, and uploaded to NVivo 12 software for coding and analysis along with the responses to the open-ended questions and organizational documentation. Quantitative data from the socio-demographic questionnaires and PSAT will be entered into SPSS 25 statistical software for analysis. We will remove the identity of participants by assigning fictitious names/codes. A familiarization stage with collected data from each strand will take place (e.g., check for trends in the quantitative data, read through qualitative data) before data analysis occurs (Creswell & Piano Clark, 2017).

### Analysis and Interpretation

We will analyze data from qualitative interviews following an adapted deductive–inductive approach developed by Damschroder and Lowery (2013). This approach has been used in several implementation efforts so that researchers and organizations can identify contextual factors that influence implementation as well as areas in need of improvement to enhance implementation outcomes. For the purposes of this study, we will code data deductively against the Consolidated Framework of Sustainability Constructs in Healthcare (CFSCH) (Lennox et al., 2018) and not the Consolidated Framework of Implementation Research (Damschroder et al., 2009) as Damschroder and Lowery (2013) did. This method of analysis (a) is a good fit with the objectives of this inquiry (i.e., identifying determinants of sustainability of recovery-oriented interventions); (b) facilitates the comparison of qualitative findings across cases; and (c) facilitates the comparison between qualitative and quantitative results based on outcomes that can be measured quantitatively.

Qualitative data analysis will include the following steps: (a) each interview will be coded deductively against the Consolidated Framework of Sustainability Constructs in Healthcare (CFSCH) (Lennox et al., 2018); (b) information that does not fit under the framework will be analyzed inductively to allow for new themes to emerge; (c) once all interviews for each site have been coded, what Damschroder and Lowery (2013) term “a case memo” will be created for each case. A case memo is a summary of the data under the CFSCH construct where interview data have been coded and illustrative quotes; (d) each case memo will undergo a rating process where each of the included constructs will be rated based on the valence (positive vs. negative) and the magnitude of its influence in sustaining the interventions. Damschroder and Lowery (2013) provide detailed instructions on the rating process; and (e) ratings across cases and constructs will be displayed in a matrix where cases will be compared and contrasted based on the level of sustainment of the recovery-oriented intervention (e.g., sustained, partially sustained, not sustained). Case memos will help contextualize the ratings.

We will analyze the responses to the socio-demographic questionnaire and the Program Sustainability Assessment Tool (PSAT) (Luke et al., 2014) using descriptive non-inferential statistics (mean, median, standard deviation). Following Stoll et al. (2015)’s steps, ES will (a) calculate site-specific means for each of the eight domains along with cross-site average scores; (b) compare sites to identify lower and higher rating statements; and (c) explore any patterns between the ratings and the level of sustainment of the intervention (i.e., sustained, not sustained, partially sustained).

Qualitative content analysis will guide the analysis of the responses to the open-ended questions for the post-implementation adaptations (Vaismoradi et al., 2013). Qualitative content analysis is a descriptive approach suitable to use in identifying trends in data that can allow for the quantification of qualitative data (Vaismoradi et al., 2013) and permitting comparison of findings within and across cases. The analysis will entail (a) participant responses from each site will be coded against FRAME; (b) subcategories or additional codes will be developed; (c) findings per case will be summarized and presented in a matrix to allow for comparison.

We will analyze organizational documents following Bowen (2009)’s recommendations for document analysis. We will read and categorize documents based on their content (e.g., progress report, budget) and identify for coding segments of texts that are pertinent to the topic of the inquiry (Bowen, 2009). Segments of texts will be coded to the constructs of the two theoretical frameworks (i.e., CFSCH, FRAME) that guide the qualitative data collection of this study along with data from interviews and written responses.

Upon completion of data analysis, we will present qualitative and quantitative results side-by side in a joint display table allowing for direct comparison and merging (Creswell & Piano Clark, 2017). The aim of integrating qualitative and quantitative findings in a convergent mixed methods design is “to develop results and interpretations that expand understanding” (Creswell & Piano Clark, 2017, p. 221). We will compare qualitative and quantitative findings looking for contradictions as well as consistencies. We will present and interpret integrated results in the discussion section (Creswell & Piano Clark, 2017).

## Trustworthiness

We will employ four strategies to establish the trustworthiness of the study. Multiple sources of data from distinct methodologies (i.e., interviews, questionnaires and documents) will be collected allowing for data triangulation (Yin, 2014). The first author will develop an electronic database per case allowing for the secure storage of collected data, accessibility, and analysis (Yin, 2014). We will maintain a detailed audit trail throughout the study where research activities, decisions, and data analysis procedures will be recorded chronologically to enhance transparency, and to track the emergence of findings from the raw data (Creswell & Miller, 2000). Finally, we are mindful that our beliefs, past experiences, and biases could shape our research. For example, the first (ES) and fifth (MPi) authors have a previous relationship with the participating sites and study participants. While this closeness could facilitate recruitment and contribute to obtaining richer data during the interviews due to the insight that authors have gained over the years, it could be also be a drawback. During data analysis, it might be difficult to separate previous knowledge of the site from what the new data indicates. In order to be transparent about these potential biases, in reporting the findings, we will reflect on our position and discuss how it could influence the research process and interpretation of the data (Creswell & Miller, 2000).

## Potential Challenges and Mitigation Strategies

Two potential challenges are foreseen in relation to the engagement of study participants: research fatigue (Clark, 2008) and staff turnover (Beidas et al., 2016; McPherson et al., 2021). In regard to the former, potential participants recently completed the 3-year initial project. Thus, their time-availability to participate in a new research effort might be limited. However, two of the authors have established a strong rapport with the participating organizations. This aspect coupled with this study’s low research burden due to its limited duration and time commitment of study participants (e.g., four hours over 15 months) along with the tangible benefits from the acquired knowledge (i.e., key factors influencing the level of sustainment of the interventions), closely related to their previous work (i.e., implementation process of recovery-oriented interventions) will facilitate the recruitment. Regarding the second challenge, we have noticed a significant turnover in managers and service providers in the sites that implemented the recovery-oriented interventions over the course of the initial project. In all four sites for this study, the majority of senior managers (three out of four) and at least one service provider in each implementation team has left their organization. This issue has also been documented in the literature both for recovery-oriented (Piat et al., 2021) and other interventions in the mental health field (Beidas et al., 2016). This may limit the number of potential participants with an in-depth knowledge of the contextual factors that influence sustainment. A mitigation strategy will be to recruit participants who have lengthier relationships with their organization as they will be more knowledgeable on the organizational structure, culture, and processes to sustain interventions.

## Discussion

Sustainability, whether it is conceptualized as an implementation outcome (Proctor et al., 2015) or process (Pluye et al., 2004), is a significant concern for all involved actors in healthcare (e.g., policy makers, funders, managers) (Proctor et al., 2015; Shelton et al., 2018). Despite this vested interest, Stirman-Wiltsey et al. (2012) in one of the first comprehensive attempts to synthesize evidence in this field, noted that the “body of literature [in sustainability] … is fragmented and underdeveloped” (p. 13). In a more recent review, Braithwaite et al. (2020) found a slight improvement on empirical research on sustainability of healthcare interventions but problems persist regarding the conceptualization of sustainability, assessment, and sporadic use of frameworks.

Researchers have not yet explored the concept of sustainability in great depth in mental health literature. This study has added value in beginning to fill this gap and it, could pave the way for more focused research. Findings from this study will impact within and beyond the research environment. From an implementation science perspective, findings will (a) expand our current evidence base on the intersection of sustainability and mental health recovery interventions that remains under-explored; (b) provide new insights into the applicability of frameworks on sustainability in accessing the capacity of organizations to sustain mental health interventions that require a radical cultural shift; (c) identify factors that influence positively or negatively their level of sustainment so implementation scientists could perhaps assess them in various implementation phases, and if necessary, take action early on; and d) advance the discussion on the post-implementation phase of mental health interventions in regards to what should be sustained and the role of adaptation as a core component of the sustainment process, what is the mechanism to achieve sustainment, and how to achieve integration into routine practice (e.g., sustainment strategies). Outside of the mental health field, knowledge generated from this study could be useful to other healthcare fields. As Lennox et al. (2018) noted, advancing our understanding of sustainability in a particular healthcare setting contributes to the development of “a sustainability knowledge base that is useful beyond specific settings or interventions” (p. 2).

In the policy front, new knowledge produced from this study could increase awareness of the importance of proactive planning for sustainability as early as in the pre-implementation phase. Policy and decision makers in Canada and elsewhere may consider developing guidelines on the sustainment of recovery-oriented interventions and practice as they plan the transformation of mental health services to a recovery-orientation approach. On the ground, organizations that are in the process of implementing mental health recovery into their services could develop a sustainment plan based on their local context and needs even before initial implementation.

We will use three strategies, based on the targeted audience, to disseminate findings (Graham et al., 2013): (a) we will inform the academic community by means of articles in peer-reviewed journals and presentations at Canadian and international conferences related to implementation and sustainability; (b) we will develop and share fact sheets (i.e., a one-page document that provides a summary of findings in lay language) with study participants across sites in an interactive online meeting to foster dialog on the sustainment of recovery-oriented interventions; and (c) we will write a brief report with sustainability strengths and weaknesses and send to participating sites to guide their sustainability planning.

There are both strengths and limitations to this study. As a case study, the findings will be pertinent to each case, as the thorough understanding of a case limits the ability to generalize the findings. Case study research allows for “particularization, not generalization” (Stake, 1995, p. 8). However, Stake (1995) also notes that after studying cases the researchers may have “assertions” or “petite generalizations” within the cases and modifications of “grand generalizations.” The proposed sample size could pose concern. However, since this is a real-world study on sustainability, the pool of potential study participants (i.e., individuals directly involved with the recovery-oriented intervention that is sustained or not) is limited. From a methodological point of view, it is a rich design combining three robust frameworks on sustainability research that will inform the collection of data from four stakeholder groups across four organizations in three Canadian provinces.
